# Uses of tranexamic acid and the risk of thrombosis in women: Number 11 – 2025

**DOI:** 10.61622/rbgo/2025FPS11

**Published:** 2025-12-16

**Authors:** Venina Isabel Poço Viana Leme de Barros, Edimárlei Gonsales Valério, Marcelo Melzer Teruchkin

**Affiliations:** 1 Universidade de São Paulo Faculdade de Medicina Hospital das Clínicas São Paulo SP Brazil Hospital das Clínicas, Faculdade de Medicina, Universidade de São Paulo, São Paulo, SP, Brazil.; 2 Universidade Federal do Rio Grande do Sul Porto Alegre RS Brazil Universidade Federal do Rio Grande do Sul, Porto Alegre, RS, Brazil.; 3 Hospital Moinhos de Vento Porto Alegre RS Brazil Hospital Moinhos de Vento, Porto Alegre, RS, Brazil.

## Key points

- Tranexamic acid (TXA) reduces the risk of bleeding and, consequently, the risk of death from hemorrhage.
- It is indicated for postpartum hemorrhage (PPH).
- It is also recommended for abnormal uterine bleeding.
- In general, TXA does not increase the risk of venous thromboembolism (VTE).
- Patients using combined oral contraceptives, who are obese or have other known risk factors for thrombosis should be individually evaluated before using TXA.

## Recommendations

- Tranexamic acid is an efficient and safe alternative in the treatment of dysfunctional uterine bleeding with a low risk of VTE.
- Although it shows good results in the management of PPH, maintaining a low risk of VTE, the thromboembolic risk must be assessed in all postpartum women.
- Tranexamic acid is effective in the prevention of PPH before cesarean sections in high-risk pregnancies.
- It is indicated for gynecological surgeries with a high risk of bleeding or established hemorrhage, minimizing the significant increase in VTE risk. However, all surgical patients should undergo thromboembolic risk assessment.
- Tranexamic acid is an option for the treatment of melasma, without increasing the risk of VTE.
- The indication of TXA for patients known to have thrombophilia should be individualized.

## Background

Bleeding events in women are very frequent, particularly during the menacme.^(1)^ Tranexamic acid (TXA) is safe and effective for the treatment of heavy vaginal bleeding during menstruation and childbirth. It improves quality of life, facilitates participation in school and work, and reduces the risk of death from postpartum hemorrhage (PPH). Although its benefits are well-established, individual and structural barriers hinder the widespread use of TXA, making effective patient care difficult and perpetuating women's health inequities.^(1)^ A meta-analysis of 216 randomized clinical trials with over 125,000 patients of different ages, genders, and medical/surgical areas showed a similar risk of thrombotic events: 2.1% in the intervention group vs. 2.0% in the control group (risk difference 0.001; 95% CI: 0.001 to 0.002), with no significant difference for VTE.

## Discussion of the topic

Tranexamic acid, an antifibrinolytic agent that competitively blocks the conversion of plasminogen to plasmin, reduces fibrinolysis by occupying lysine binding sites.^(2)^ Despite theoretical concerns about hypercoagulability, a clear distinction exists between pro-hemostatic and pro-thrombotic effects. This medication has been used since 1962 in PPH in Japan, reduces morbidity and mortality, and is also indicated for heavy menstrual bleeding and for patients with abnormal uterine bleeding (AUB) when hormone therapy is contraindicated.^(2)^ Clinical trials show that its efficacy and safety vary according to context, timing and dose.^(2,3)^ Chart 1 shows the main indications and dosages.

## What is the role of tranexamic acid in PPH?

The WOMAN study showed that TXA reduces bleeding deaths in patients with PPH by 30% without increasing adverse effects.^(10)^ It should be administered rapidly after the onset of bleeding, along with other treatments, as delays decrease the benefit by 10% every 15 minutes up to three hours; after this time, there is no advantage. The recommended dose is 1 g intravenously over 10-20 minutes, which can be repeated after 30 minutes if bleeding continues.^(11)^

The antifibrinolytic effect of TXA persists in serum for seven to eight hours. The concentration of the substance in breast milk corresponds to approximately one hundredth of the maximum serum concentration, therefore it is unlikely that any antifibrinolytic effect is exerted in the newborn.

A randomized, double-blind, placebo-controlled study involving 193 hospitals in 21 countries evaluated the early impact of intravenous TXA on mortality, hysterectomy, and other complications in over 20,000 patients with diagnosed PPH.^(11)^ Patients were eligible for randomization if blood loss was > 500 mL in vaginal delivery, > 1,000 mL in cesarean section, or associated with hemodynamic instability. All other aspects of PPH management followed usual standards. Approximately 70% of deliveries were vaginal and 30% were cesarean sections. Compared with placebo, the use of TXA:

- promoted a 19% reduction in death due to bleeding overall (1.5% vs. 1.9%; relative risk [RR]: 0.81; 95% CI: 0.65-1.00)
- reduced mortality from bleeding observed after both vaginal deliveries and cesarean sections. Death due to hemorrhage was reduced by 31% when treatment was initiated within three hours of birth (1.2% vs. 1.7%; RR: 0.69; 95% CI: 0.52-0.91) and by 26% for hemorrhage due to atony (1.2% vs. 1.6%; RR: 0.74; 95% CI: 0.55-0.99). In contrast, the reduction was not significant when the time after birth was greater than three hours and in patients with other causes of bleeding or unknown causes;
- reduced the incidence of laparotomy for bleeding control by 36% (0.8% vs. 1.3%; RR: 0.64; 95% CI: 0.49-0.85);
- did not reduce overall mortality, including causes such as sepsis, organ failure, eclampsia, or pulmonary embolism, which accounted for more than 25% of deaths. Only death from bleeding showed a significant change;
- did not increase the risk of VTE.^(10)^

Two additional meta-analyses of randomized trials have demonstrated that TXA reduces bleeding-related mortality in patients with primary PPH, regardless of the type of delivery.^(4,5)^ However, the use of TXA in patients with moderate to severe anemia did not prevent PPH.

## What is the role of TXA in AUB?

Tranexamic acid is approved by the Food and Drug Administration (FDA) for the treatment of AUB. It is indicated in cases of heavy menstrual bleeding not controlled by other medications. It is also commonly used to treat heavy menstrual bleeding in women with bleeding disorders, especially von Willebrand disease.^(12)^

Another use option is the treatment of patients with AUB in whom hormone therapy is contraindicated (e.g., personal history of breast cancer). However, its role in patients with an increased risk of thrombosis is controversial.^(12)^

A recent Cochrane review suggests that the use of levonorgestrel intrauterine systems (LNG-IUS) is the best first-line treatment to reduce menstrual blood loss. Antifibrinolytics are probably the second best treatment, and long-cycle progestogens the third best treatment.^(13)^

## What is the evidence of efficacy of TXA in AUB?

Tranexamic acid has proven to be an effective treatment for the treatment of AUB as it reduces menstrual blood loss by 26% to 60%.^(6,7)^ The frequency of the most common adverse effects (menstrual cramps, headache, back pain, and nausea) was similar in both groups.

## What is the dose and duration of treatment with TXA in AUB?

The recommended dose is 1,300 mg (two 650 mg tablets) three times a day for five days during the menstrual period. It acts within two to three hours after administration.^(7)^ In Brazil, 250 mg and 500 mg tablets are available.

## What is the risk of VTE with the use of TXA in AUB?

Tranexamic acid is generally well tolerated. Some studies do not show a significantly increased risk of VTE with the use of TXA. A case-control study conducted in Sweden did not demonstrate an increased risk of VTE in patients using TXA.^(5)^ Sundström *et al*.^(4)^ suggest that anemia associated with AUB represents a pro-thrombotic condition.

In a literature review, Thorne *et al*.^(6)^ concluded that the use of TXA is reasonable in patients using combined hormonal contraceptives, provided that additional risk factors for thrombosis (obesity, immobility, coagulopathy) are not present.

## Can TXA be used in the third stage of labor for PPH prophylaxis?

- Use in vaginal deliveries - In a meta-analysis of randomized clinical trials (four trials, 4,671 participants), the prophylactic use of TXA after vaginal delivery, compared with placebo, did not significantly reduce the transfusion rate (0.8% vs. 1.0%; RR: 0.87; 95% CI: 0.46-1.64), but also did not increase the risk of thrombotic events.^(14)^
- Use in cesarean sections – Meta-analyses of randomized clinical trials of prophylactic use of TXA after cesarean sections reported a reduction in blood loss. However, these are meta-analyses lacking high-quality evidence, so their results should be viewed with caution.^(15,16)^ A 2023 meta-analysis of randomized clinical trials compared the prophylactic use of TXA with placebo, no treatment, standard treatment, or prostaglandin analogue controls in cesarean delivery.^(17)^ In this meta-analysis, prophylactic TXA demonstrated a reduction in total blood loss, and this difference was greater in the high-risk patient group and when administered at the skin incision compared to administration after cord clamping. There are important factors to consider in this meta-analysis: the two largest trials^(15,16)^ did not report substantial benefits of TXA in reducing the risk of PPH in a largely low-risk population, directly contrasting with the numerous smaller trials that reported significant reductions in blood loss. In a systematic review of meta-analyses of randomized studies including 2,365 patients at low and high risk for PPH^(8)^ the administration of 1 g of TXA intravenously over a period of 10 to 20 minutes before skin incision was significantly more effective than placebo in reducing bleeding volume, transfusions, hemoglobin levels, as well as the need for hemorrhage interventions. A similar study with patients at high risk for PPH undergoing cesarean section showed a significant reduction in blood loss (50%).^(9)^ The number of thromboembolic events was very low and not significant between the two groups.^(8)^

Although TXA freely crosses the placenta, no fetal harm has been reported in the available evidence, and it appears to be more effective if administered at the beginning of the cesarean section (before incision).^(8)^

## Can ATX be used in gynecological surgery?

Knowledge about intraoperative TXA administration and the increased risk of thromboembolic events is limited.^(1)^ Decisions regarding the use of TXA in patients considered to be at higher risk for thrombotic events should be individualized, taking into account the bleeding and transfusion risks related to the patient and the surgical procedure.

A systematic review that included 216 randomized trials of intravenous TXA vs. placebo in 125,550 surgical and non-surgical patients found no increased risk of thromboembolism in studies that included patients with a history of thromboembolism.^(18)^ Overall, the dose of intravenous TXA administration varied widely, from 0.5 to 5 g or 10 to 100 mg/kg. The shift from a single-dose approach to weight-adjusted dosing may be associated with the different treatment regimens applied worldwide in the context of trauma and surgery. The authors did not detect any dose-dependent association of adverse events.^(18)^ Similar results were found in a recent review of obstetric and gynecological surgery patients.^(19)^

## Can TXA be used in the treatment of melasma?

Melasma is a hyperpigmentation and vascularization disorder frequently found in women aged between 20 and 40 years. Although melasma is usually treated with topical hydroquinone, it is often refractory to treatment. Tranexamic acid is a plasmin inhibitor used off-label in the treatment of melasma and can be administered orally, topically, or intralesionally (by injection).^(18,20)^

Tranexamic acid is an option for this difficult-to-treat disease. The average dose in the treatment of melasma is 250 mg twice a day. Although the dose used in clinical studies for the treatment of melasma is considerably lower than that used to treat bleeding conditions, patients should be evaluated for thrombotic risk.^(21-23)^

## What are the most common adverse effects with the use of TXA?

Nausea, vomiting, diarrhea, and abdominal pain may occur in 20% of patients. These events improve with oral ingestion of the tablets with food. The following may occur: headache in 50% of patients; muscle pain, arthralgia, and back pain in 10%; fatigue in 5%; visual disturbances such as blurred vision, hazy vision, or altered color vision in less than 1%; seizure in less than 1% of patients (associated with higher doses and in patients with liver disease).^(1,24)^

## What are the contraindications and precautions with TXA?

Contraindications include: known hypercoagulability conditions;^(24)^ patients with disseminated intravascular coagulation, since inhibition of the fibrinolytic system may increase the risk of thrombotic complications in this setting.^(24)^ The dose of TXA should be reduced in cases of renal insufficiency.^(24)^

## Final considerations

in conclusion, TXA is an effective and safe option in the treatment of bleeding in women, including heavy menstrual bleeding, PPH, and in gynecological surgeries with a low risk of thromboembolic complications. In patients with medium and high risk of VTE, its use should be individualized.

## National Commission Specialized on Venous Thromboembolism and Hemorrhage in Women of the Brazilian Federation of Gynecology and Obstetrics Associations (Febrasgo)

President

Venina Isabel Poco Viana Leme de Barros

Vice-President

Egle Cristina Couto

Secretary

Joaquim Luiz de Castro Moreira

Members

Edimárlei Gonsales Valerio
  Juliana Silva Esteves
  Paulo Francisco Ramos Margarido
  Ana Maria Kondo Igai
  Cristiano Caetano Salazar
  Dênis José Nascimento
  Eduardo Zlotnik
  Fernanda Andrade Orsi
  Marcelo Melzer Teruchkin
  Marcos Arêas Marques
  Mônica Cristina da Costa Drago Souza
